# Airway resistance and reactance are affected in systemic sclerosis

**DOI:** 10.3402/ecrj.v2.28667

**Published:** 2015-10-21

**Authors:** David Aronsson, Roger Hesselstrand, Gracijela Bozovic, Dirk M. Wuttge, Ellen Tufvesson

**Affiliations:** 1 Respiratory Medicine and Allergology, Department of Clinical Sciences Lund, Skåne University Hospital, Lund University, Lund, Sweden; 2 Rheumatology, Department of Clinical Sciences Lund, Skåne University Hospital, Lund University, Lund, Sweden; 3 Diagnostic Radiology, Department of Clinical Sciences Lund, Skåne University, Hospital, Lund University, Lund, Sweden

**Keywords:** fibrosis, impulse oscillometry, reactance, resistance, systemic sclerosis

## Abstract

**Background:**

Interstitial lung disease often occurs as an early complication of systemic sclerosis (SSc). The aim was to investigate whether impulse oscillometry (IOS) could be used to evaluate lung impairment in SSc.

**Methods:**

Seventy-eight SSc patients, of which 65 had limited cutaneous SSc (lcSSc) and 13 had diffuse cutaneous SSc (dcSSc), were subjected to high-resolution computed tomography (HRCT) and pulmonary function tests (spirometry, IOS, and single breath CO diffusion capacity test). Twenty-six healthy individuals served as controls.

**Results:**

Patients with lcSSc had higher levels of peripheral airway resistance, that is, R5–R20 (difference between resistance at 5 Hz and resistance at 20 Hz) showed a median (and interquartile range) of 0.05 (0.02–0.09) in lcSSc, 0.01 (0.00–0.04) in dcSSc and 0.04 (0.01–0.06) in healthy controls. They also had higher levels of reactance: reactance area was 0.26 (0.15–0.56) in lcSSc, 0.20 (0.11–0.29) in dcSSc and 0.18 (0.08–0.30) in healthy controls, and resonant frequency was 10.9 (8.8–14.8) in lcSSc, 9.0 (8.3–11.6) in dcSSc and 9.1 (8.0–13.1) in healthy controls. Airway reactance correlated to fibrotic findings on HRCT, such as ground glass opacities and reticulations.

**Discussion:**

This implies that IOS parameters to some extent are related to fibrosis in patients with SSc.

Interstitial lung disease (ILD) is a severe complication of systemic sclerosis (SSc). The unfavourable clinical outcome of pulmonary complications underlines the importance of early identification of patients in potential need of treatment with immunosuppressive agents. Conventional pulmonary function evaluation together with high-resolution computed tomography (HRCT) analysis of the lungs is currently a standard measure of SSc-ILD (1). However, development of analysis of lung involvement by non-invasive methods is needed to better stratify patients for treatment in clinical praxis.

Impulse oscillometry (IOS) is a non-invasive forced oscillation technique used to evaluate airway resistance and reactance. Unlike conventional methods of pulmonary function testing (e.g. spirometry), IOS requires minimal cooperation from the patients. The forced oscillations are superimposed on the normal breathing and avoid the need for any special breathing manoeuvre or any noticeable interference with respiration. IOS has been used to assess the peripheral airway involvement in mainly asthma and chronic obstructive pulmonary disease (2, 3). In SSc, ILD always occurs very early (4), and the major deterioration of lung function occurs during the first 4 years after disease onset (5). It has previously been shown that SSc patients with a spirometric restrictivity show a changed pattern of resistance and reactance using forced oscillation technique (6). We therefore aimed to further investigate whether IOS could be used to evaluate lung impairment in SSc patients with short disease duration, or could be useful in predicting lung function impairment. The aim was to investigate resistance and reactance in SSc patients compared to healthy controls, and relate to radiological findings of SSc-ILD.

## Methods

### Patients

Seventy-eight patients who fulfilled criteria for SSc according to the 2013 classification criteria for systemic sclerosis (7) were included. Sixty-five of these patients had limited cutaneous SSc (lcSSc) and 13 had diffuse cutaneous SSc (dcSSc). Twenty-six healthy non-smoking individuals with no history of respiratory diseases served as controls (Table 1).

**Table 1:** Subject characteristics

	Healthy subjects (*n=*26)	lcSSc (*n=*65)	dcSSc (*n=*13)	
Age, years	53 (50–57)	60 (50–68)	56 (42–66)	n.s.
Females/males, *n*	17/9	52/13	8/5	n.s.
Smokers, *n*	0	8	1	n.s.
Disease duration, years	n.a.	3.2 (1–7.8)	1.0 (0.6–1.0)	**
Ground glass opacity, %	n.d.	0 (0–15)	5 (0–15)	n.s.
Reticulations, %	n.d.	0 (0–10)	5 (0–20)	n.s.
Ground glass opacity/reticulations	n.d.	0 (0–10)	5 (0–15)	n.s.
Traction bronchiectasis score	n.d.	0 (0–2)	2 (0–2)	n.s.
VC, %p	99 (93–106)	92 (80–103)	82 (67–94)	**
FEV1, %p	98 (87–108)	88 (80–99)	85 (65–100)	*
DLCO, %p	n.d.	78 (57–91)	65 (53–72)	n.s.
DLCO/VA, %p	n.d.	67 (49–78)	56 (45–62)	n.s.
FeNO50, ppb	18.9 (13.0–29.1)	16.7 (12.7–26.6)	19.7 (7.8–30.5)	n.s.
Alveolar NO, ppb	2.1 (1.6–2.6)	3.4 (2.7–4.2)	3.7 (2.3–4.7)	***
Bronchial NO flux, nl/sec	0.9 (0.6–1.4)	0.8 (0.6–1.3)	0.9 (0.3–1.6)	n.s.

%p=% of predicted normal; DLCO=diffusion lung capacity of CO; FeNO=fractional exhaled nitric oxide; FEV1=forced expiratory volume in 1 sec; VA=alveolar volume; VC=vital capacity; n.a.=not applicable; n.d.=not determined; n.s.=non-significant and

*,**,*** (*p*<0.05, <0.01, and <0.001, respectively) depict statistically significance over several groups (Kruskal–Wallis).

### Study design

All patients were investigated when admitted for assessment of SSc. Spirometry, IOS, single-breath carbon monoxide diffusion (DLCO), fractional exhaled nitric oxide (FENO) (calculating alveolar nitric oxide concentration) and HRCT were performed. At a follow-up visit after 1 year (in 61 of the SSc patients), spirometry, IOS, DLCO and FENO were performed. All subjects gave written informed consent, and the regional ethics review board in Lund approved the study.

### Lung function tests

All subjects performed IOS (Jaeger MasterScreen, Erich Jaeger GmbH, Würzburg, Germany) and flow-volume spirometry (Jaeger MasterScreen), and the SSc patients also performed single breath helium dilution with CO-diffusion capacity test due to clinical routine (Jaeger MasterScreen Diffusion). Reference values from Crapo et al. (8) were used for spirometry, and from ECCS (9) for DLCO.

IOS was performed for about 30 sec. Oscillometric pressure impulses were superimposed on the tidal breathing of the subject, having a pulse sequence of 5 per second and a frequency spectrum between 5 and 35 Hz. The subjects used nose clips and were told to press the palms of their hands against the cheeks to decrease the upper airways shunt.

The IOS system produces a pulse-shaped pressure wave, sending it down the subject's lungs. The corresponding flow is then recorded to determine respiratory impedance. The oscillations provide a measure of total airway impedance, which reflects both resistive elements of the airways (resistance, R) and viscoelastic and inertive forces in the lungs and the chestwall (reactance, X). Reactance is the sum of inertance, which is the inertive force of the air column in the conducting airways, and capacitance, which reflects the elastic properties of the peripheral lung. Inertive forces dominate at frequencies above resonant frequency (Fres), whereas elastic forces are increasingly related to frequencies below Fres. Low frequent reactance is usually reported as X5 (reactance at 5 Hz). X5 reflects changes to the lung periphery and is non-specific. Increased negative values can be seen in both restrictive and obstructive diseases. Resonant frequency is the frequency where the inertance and capacitance are equal in magnitude and opposite in sign (phase), and are measured in Hz. The reactance area (AX) is a quantification of the respiratory reactance between 5 Hz and Fres; thus, it will mostly reflect the capacitance part of the reactance. This makes AX a good index of changes of the degree of peripheral involvement.

Resistance is typically reported as R5 and R20 (resistance at 5 and 20 Hz, respectively), where R5 reflects total respiratory resistance of the airways and R20 reflects resistance of the proximal airways. The fall in resistance from R5 to R20 (=R5 - R20) was used as a surrogate for the frequency independence of respiratory resistance, which increases with increasing inhomogeneity of peripheral airways. The R5 - R20 parameter is thereby suggested to be an indicator of peripheral properties of the respiratory tract (3).

### Measurement of fractional exhaled nitric oxide

NO measurements were performed using a NIOX (Aerocrine, AB, Stockholm, Sweden) as described previously (10). Patients were comfortably seated. They inhaled NO-depleted ambient air and exhaled at different flow rates [50 (giving FENO50), 100, 200, and 400 ml/sec] 3–4 times, depending on divergence. Alveolar NO concentration and bronchial NO flux were approximated by plotting NO output (production of concentration and flow) against exhaled flow (at 100–400 ml/sec), and were approximated by the slope and intercept of this line, respectively (11, 12).

### High-resolution computed tomography

The HRCT images were analysed by a chest radiologist (GB) blinded for clinical data as previously described (10). The lungs were divided into six parts using the carina and pulmonary venous confluence as landmarks, surveying the distribution of changes. Each part was analysed for the extent of ground glass opacities, reticulations, and combined ground glass opacities and reticulations finally estimated in the percentage of the total lung volume with a minimum step size of 5%. Further, the presence of traction bronchiectasis, honeycombing, and emphysema were quantified in a four-step scale (none, subtle, moderate, and severe). Fibrosis was defined by the presence of combined ground glass opacities and reticulation (13) strongly confirmed with the presence of traction bronchiectasis within these areas (14).

### Statistics

Kruskal–Wallis non-parametric test was used for trend analyses between several groups. Mann–Whitney test was used for statistical comparison between separate groups and chi-square test was used for nominal data. Wilcoxon test was used for statistical comparison before and after 1-year follow-up. Correlations were analysed using Spearman's non-parametric correlation test. All statistical analyses were done using GraphPad Prism 5.0 and a *p*<0.05 was considered significant. All data were presented as median (interquartile range).

## Results

**Fig. 1:**
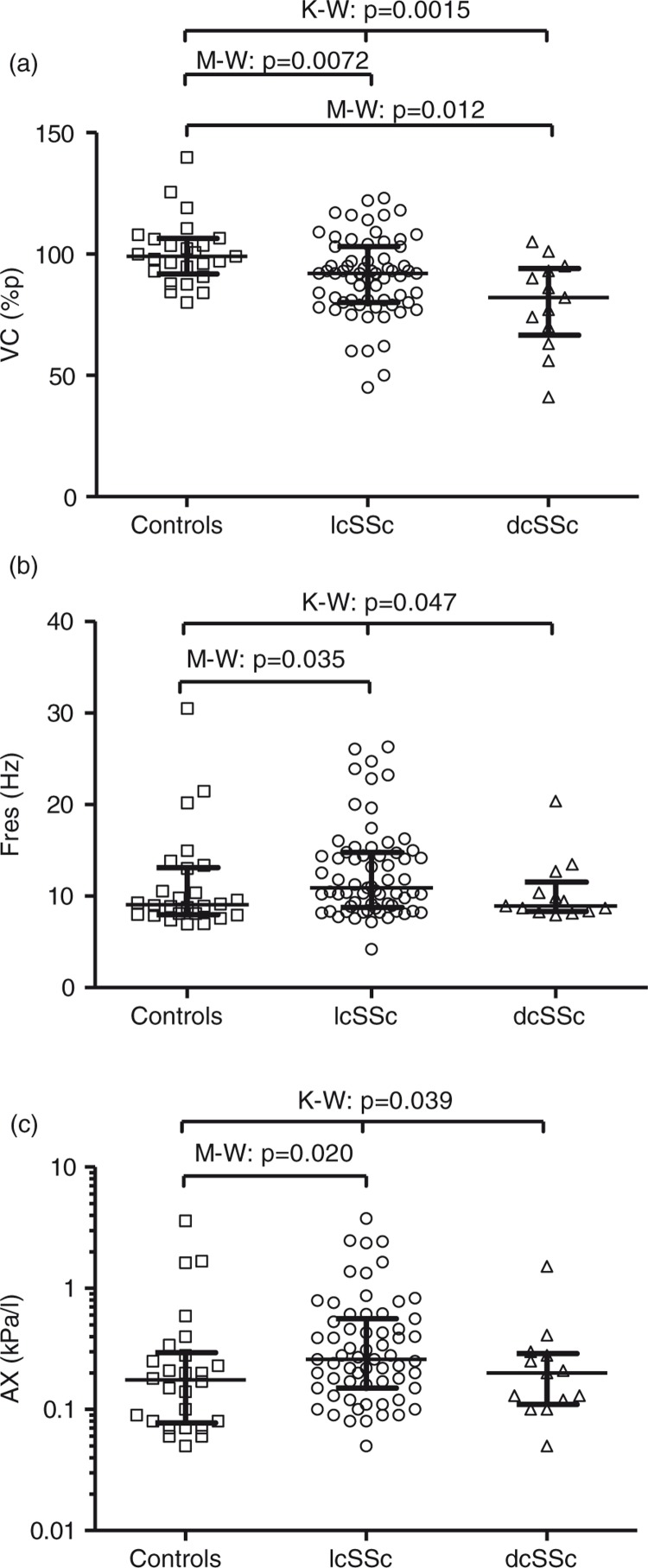
(a) Vital capacity (%p), (b) resonant frequency (Fres), and (c) reactance area (AX) for patients with lcSSc, dcSSc, and healthy controls. Data are presented as individual scores and median with interquartile range. Kruskal–Wallis (K–W) test was used for trend analyses among several groups, and Mann–Whitney (M–W) test was used for statistical comparison between separate groups.

Both patients with lcSSc and patients with dcSSc had lower vital capacity (VC, % of predicted) and forced expiratory volume in 1 sec (FEV_1_, % of predicted) than healthy controls (Fig. 1a and Table 1), showing an overall lung function impairment among the SSc patients as expected. In addition, IOS measurements showed increased peripheral airway involvement, specifically airway reactance, in the group of patients with lcSSc, compared to healthy controls [reactance area (AX): *p=*0.020 and resonance frequency (Fres): *p=*0.035; Fig. 1b and c]. No significant difference was seen in airway resistance at 5 Hz (R5) or 20 Hz (R20), but a variance among the groups (*p=*0.029) in the difference between R5 and R20 (=R5 - R20), which is suggested to reflect resistance in the peripheral airways (Table 2).

**Table 2:** Impulse oscillometry parameters in SSc patients and healthy subjects

	Healthy subjects (*n=*26)	lcSSc (*n=*65)	dcSSc (*n=*13)	
R5, kPa*s/l	0.38 (0.28–0.46)	0.37 (0.27–0.46)	0.32 (0.28–0.43)	n.s.
R20, kPa*s/l	0.33 (0.26–0.40)	0.32 (0.25–0.38)	0.30 (0.23–0.38)	n.s.
R5–R20, kPa*s/l	0.04 (0.01–0.06)	0.05 (0.02–0.09)	0.01 (0.00–0.04)	*
X5, kPa*s/l	−0.09 (−0.12–0.06)	−0.10 (−0.16–0.07)	−0.10 (−0.15–0.08)	n.s.
Fres, l/sec	9.1 (8.0–13.1)	10.9 (8.8–14.8)	9.0 (8.3–11.6)	*
AX, kPa/l	0.18 (0.08–0.30)	0.26 (0.15–0.56)	0.20 (0.11–0.29)	*

R5=resistance at 5 Hz, R20=resistance at 20 Hz; X5=reactance at 5 Hz; Fres=resonant frequency; AX=reactance area; n.s.=non-significant and *p<0.05 depict statistically significance over several groups (Kruskal–Wallis).

In addition, there were relations between IOS parameters reflecting the peripheral airways and radiographic quantification of ILD (all SSc patients included). Most apparent were associations to AX, which was related to ground glass opacity (*p=*0.045, *r=*0.21) and reticulations (*p=*0.022, *r=*0.23). Fres was similarly correlated to reticulations (*p=*0.035, *r=*0.21). Peripheral resistance (measured as R5 - R20) also showed a tendency to correlate to reticulations (*p=*0.056, *r=*0.18).

The alveolar nitric oxide concentrations were measured as a biomarker for inflammation in the alveolar compartment, and both AX (*p=*0.003, *r=*0.30) and Fres (*p=*0.025, *r=*0.23) were associated with the levels of exhaled alveolar nitric oxide.

There were no significant changes in lung function (spirometry and IOS) at the 1-year follow-up in comparison to the first visit. Neither did any increased IOS parameters at inclusion visit predict any changes in lung function (FEV_1_, DLCO, or exhaled alveolar nitric oxide concentration) in SSc patients at follow-up after 1 year.

## Discussion

There was a significant increase in both resistance and reactance (such as AX and Fres) measured by IOS in the group of lcSSc patients compared to healthy controls. Unexpectedly, no increase in IOS parameters could be detected in patients with dcSSc even though this group of patients often develops internal organ involvement, such as ILD. Though, this group had impaired VC and FEV_1_, showing some sort of lung engagement that is obviously not detectable by IOS.

The absolute differences in medians between the groups were very small and there was an overlap of individual values. The clinical value of using IOS as a sensitive tool for picking up early inflammatory activity and fibrotic changes in SSc is therefore not satisfactory. We expected IOS to more clearly reveal ILD in SSc patients, but so was not the case and the reason for this is unclear. In SSc-associated ILD, the most common pathologic patterns are non-specific interstitial pneumonitis (NSIP) and usual interstitial pneumonitis (UIP) (15). Key histological features in NSIP and UIP are expansion of the alveolar septa, interstitial infiltration of mononuclear inflammatory cells and varying degrees of interstitial fibrosis. In both NSIP and UIP, the fibrotic changes typically have a peripheral subpleural distribution (16). Possibly, the fibrotisation of alveolar parenchyma in SSc is located very distal in the airway tree and on the boundary to be detected by IOS. We also had very few patients with severe ILD, and it is possible that we would have seen more pronounced differences in resistance and reactance in patients with more lung engagement.

Our hypothesis was also that resistance and reactance measured by IOS were related to early changes in primary lung inflammation in SSc patients. Both AX and Fres were associated with levels of exhaled alveolar nitric oxide, which are in accordance with our previous study on asthmatics (12). We have previously also shown that alveolar nitric oxide concentration is related to early onset of ILD in SSc (10), which strengthens the relation between IOS parameters and ILD in SSc identified in the present study.

We could, however, not show that IOS can be used to predict lung impairment after 1 year. It might be that 1 year is too short time, since we could not find any significant difference in lung function among the patients. A longer time until follow-up could explore the question better and show the importance of IOS measurements.

Conclusively, there was an increase in reactance and resistance in the peripheral airways in SSc patients. Airway reactance also correlated to ground glass opacity, which may suggest that earlier or unspecific pathologies could be reflected by IOS. This demonstrates that the pulmonary fibrosis in SSc was to some extent measurable by IOS.
